# Le lymphangiome kystique du plancher buccal étendu a la région sous mandibulaire de l’adulte

**DOI:** 10.11604/pamj.2016.24.202.8395

**Published:** 2016-07-08

**Authors:** Elboukhari Ali, Nador Karim, Attifi Hicham, Zalagh Mohamed

**Affiliations:** 1 Service d’Otorhinolaryngologie et de Chirurgie Cervico-faciale, Hopital Militaire Moulay Ismail, Meknès, Maroc

**Keywords:** Cystic hygroma, lymphangiome kystique, cavité orale, Cystic hygroma, cystic lymphangioma, oral cavity

## Abstract

Les lymphangiomes kystiques sont des lésions congénitales bénignes prédominantes nettement au niveau de la région de la tête et du cou plus particulièrement dans le triangle cervical postérieur. Ces malformations des vaisseaux lymphatiques sont fréquemment diagnostiquées chez l'enfant. Nous rapportons le cas clinique d'un lymphangiome kystique du plancher buccal ayant survenu chez un adulte de 28 ans et qui s'est étendu progressivement vers la région sous mandibulaire gauche. La tumeur suspecté cliniquement a été bien exploré à la tomodensitométrie. Le traitement a consisté en une exérèse complète par voie endobuccale avec étude histologique qui a confirmé le diagnostic d'un lymphangiome kystique macroscopique. Le suivi à quatre ans n'a montré aucune récidive.

## Abstract (en)

Cystic lymphangiomas are congenital benign lesions occurring predominantly in the head and neck area, especially in the posterior cervical triangle. These malformations involving the lymphatic vessels are frequently diagnosed in children. We report the clinical case of a 28-year-old adult patient with cystic lymphangioma on the floor of the oral cavity extending gradually to the left submandibular region. The clinically suspected tumor was thoroughly explored using tomodensitometry. The treatment consisted of complete resection, endo-orally performed, associated with histological examination confirming the macroscopic diagnosis of cystic lymphangioma. Follow-up at 4 years showed no recurrence.

## Introduction

Le lymphangiome est une malformation congénitale bénigne des vaisseaux lymphatiques vu presque exclusivement chez l'enfant de moins de deux ans. Il est extrêmement rare chez l adulte. Ces malformations sont souvent retrouvées au niveau de la tête et du cou. Dans la cavité buccale, la langue est le site le plus fréquent du lymphangiome, cependant cette lésion est exceptionnellement rapportée au niveau du plancher buccal [1]. Il est considéré comme une séquestration du tissu lymphatique qui a conservé son potentiel de croissance. Trois variantes ont été décrites pour ces lymphangiomes [1, 2]: le lymphangiome capillaire, caverneux et kystique. Nous rapportons le cas d'un lymphangiome kystique du plancher buccal étendu jusqu’ à la région sous mandibulaire gauche ayant survenu chez un adulte jeune.

## Patient et observation

**Figure 1:**
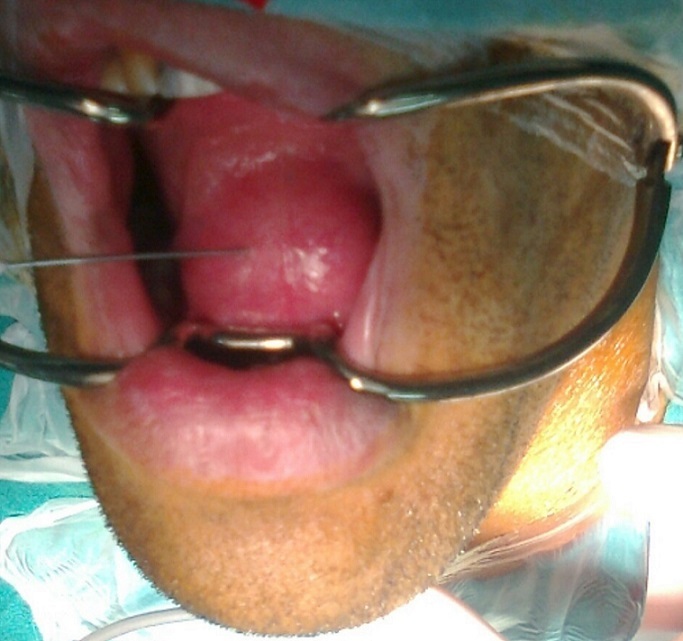
Lymphangiome kystique du plancher buccal saillant au niveau de la cavité buccale

**Figure 2:**
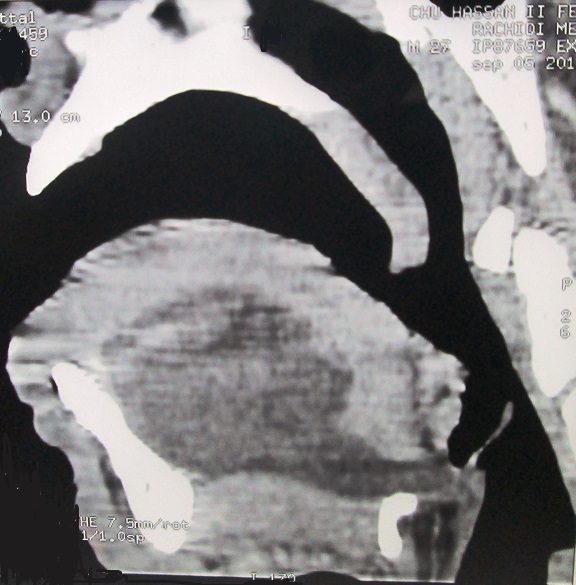
Tomodensitométrie du plancher buccal en coupe sagittale révélant un lymphangiome kystique étendu jusqu’au larynx

**Figure 3:**
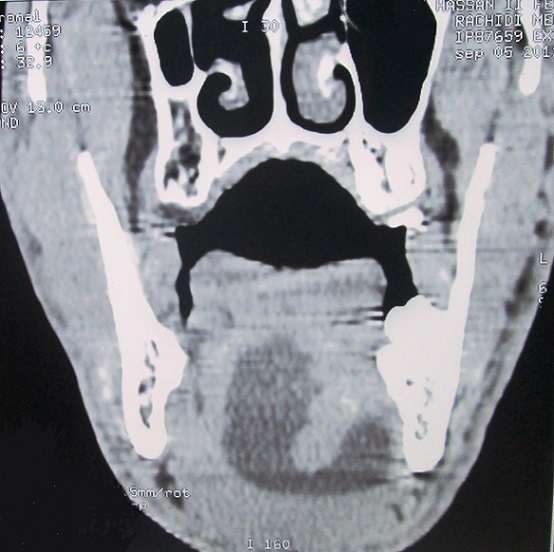
Tomodensitométrie du plancher buccal en coupe coronale révélant un lymphangiome kystique étendu vers la loge sous maxillaire gauche

**Figure 4:**
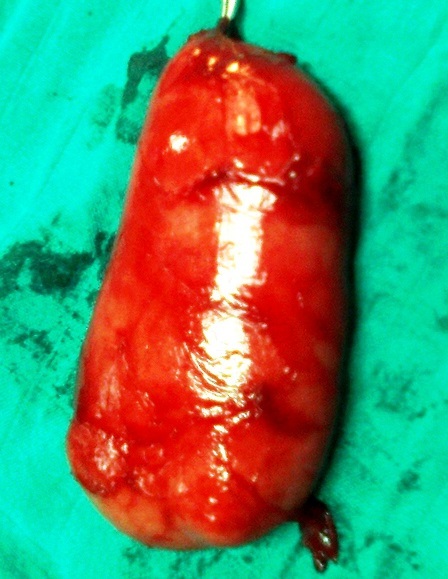
Pièce opératoire d’un lymphangiome kystique du plancher buccal

Il s'agit d'un homme de 28 ans, sans antécédents pathologiques particuliers, dont la symptomatologie a débuté il y a un ans par l'apparition progressive d'une tuméfaction du plancher buccal, isolée au début, qui a augmenté insidieusement de volume, indolore, rénitente, sans signe inflammatoire de la peau et de la muqueuse en regard, transilluminante, sans signe compressif, avec légères difficultés masticatoires (Figure 1). 6 mois plutard environ, la tuméfaction a débordé vers le bas les limites du plancher buccal pour s'insinuer entre ses muscles et passer en partie dans la loge sous mandibulaire gauche. Cette dernière est devenue tuméfiée, rénitente et molle, dont la pression se traduisait par une expansion vers le haut de la muqueuse du plancher buccal. La tomodensitométrie cervicale a confirmé la nature kystique de la tuméfaction siégeant au niveau du plancher buccal qu'il occupe en totalité se continuant en arrière vers le larynx et en bas et latéralement à travers le hiatus musculaire dans la région sous mandibulaire gauche (Figure 2) et (Figure 3). Le contenu de cette tuméfaction est hypodense, homogène, bien limité, non cloisonné et uniloculaire. Le traitement de la tuméfaction kystique a consisté en une exérèse sous anesthésie générale par voie endobuccale. En effet à travers une incision longitudinale de la muqueuse du plancher buccale, la formation kystique a été disséquée soigneusement des parois du plancher buccal; La partie sous mandibulaire a été extériorisé de sa loge vers le plancher à travers le hiatus musculaire. La pièce opératoire extraite est de nature kystique, de forme ovalaire à paroi épaisse, de surface régulière et à limites nettes; elle mesurait 76 x 40 x 37 mm, formant une cavité kystique unique contenant un liquide claire et visqueux (Figure 4). L’étude histopathologique de la pièce opératoire a posé le diagnostic d'un lymphangiome kystique. Le patient est sorti du service au troisième jour de l'intervention. La surveillance régulière n'a révélé aucune récidive avec un recul de quatre ans.

## Discussion

Les lymphangiomes sont des lésions bénignes et congénitales du système lymphatique liées très probablement à une séquestration aberrante des tissus et ou des vaisseaux lymphatiques survenant au cours de la phase de développement embryonnaire, donnant alors des voies lymphatiques bloquées; ces dernières subissent une expansion progressive sous pression hydrostatique du liquide lymphatique jusqu’ a ce qu'un équilibre avec les tissus environnants est atteint [3, 4]. 65 à 75 % des lésions sont présentes dés la naissance, et 80 à 90 % sont diagnostiquées vers l'âge de trois ans, leur survenue chez les adultes est très rarement constaté [4, 5]. En raison d'un vaste système lymphatique, la région cervicale représente le site privilégié de ces lésions qui siègent dans 75 à 80% des cas dans le triangle postérieur [5]. Cependant ces lymphatiques peuvent siéger dans n'importe quelle région du corps. Au niveau de la cavité buccal, la langue est le siège principal de ces lésions, par ailleurs le plancher buccal est très rarement concerné. Ces lymphangiomes sont généralement classés en: lymphangiomes capillaires, qui sont composés de petits vaisseaux lymphatiques à parois minces; lymphangiomes caverneux composés de vaisseaux lymphatiques dilatés entourés d'un adventice et lymphangiomes kystiques communément appelé hygroma kystique constitué d'énormes espaces lymphatiques [6]. Ces lymphangiomes kystiques peuvent êtres classés selon la taille de leur kystes et on distingue: le lymphangiome microkystique qui est composé de kystes de moins de deux centimètre de volume; le lymphangiome macrokystique avec des kystes de plus de deux centimètre et le lymphangiome mixte dont les kystes sont de taille variable [3].

Le lymphangiome kystique peut être asymptomatique apparaissant sous forme d'une tuméfaction molle, rénitente, régulière et bien limitée ou se manifestant à la suite de complications en rapport avec la compression et le refoulement des structures de voisinage: des signes respiratoires, dysphagie, troubles neurologiques périphériques, surinfection avec fistulisation à la peau ou hémorragies intrakystique [7]. La nature kystique de la tumeur est suspectée sur l'examen clinique, doit être confirmée par les données de l’imagerie [8]. L'échographie est l'examen de première intension permet de montrer la tumeur kystique sous forme d'une masse hypo-échogène bien limitée; elle est utile pour le diagnostic prénatal des lymphangiomes kystiques. La tomodensitométrie montre une masse de contenu hypodense, bien circonscrite sans invasion des structures anatomiques environnantes. L’IRM est complémentaire au scanner permet d'étudier les rapports du lymphangiome avec les structures de voisinage. L'imagerie permet également de faire le diagnostic différentiel avec d'autres lésions cervico-faciales de nature kystique, tel que: le kyste bronchial, le kyste thymique, le kyste du tractus thyreoglosse, l'abcès collecté, l'hématome, les tumeurs nécrosées, le tératome kystisé [8]. Le traitement des lymphangiomes kystiques de la cavité buccale devient parfois obligatoire voir urgent devant la présence de complications; Il fait essentiellement appel à la chirurgie qui consiste en une exérèse complète du kyste afin d'éviter les récidives [4, 9]. Les autres alternatives thérapeutiques devant un lymphangiome kystique simple sont: Le drainage-aspiration, les stéroïdes, la sclerothérapie, l'exérèse au laser, l'ablation et la cautérisation à la radiofréquence, la radiothérapie utilisée il y a longtemps est actuellement abandonnée [3, 4].

## Conclusion

Le lymphangiome kystique est une tumeur bénigne connue habituellement par sa survenue à un âge très précoce. Il est plus fréquemment rencontré au niveau cervical. Cette tumeur est exceptionnellement décrite chez un adulte au niveau du plancher buccal. En assurer un bon pronostic, fait appel essentiellement à la chirurgie avant la survenue de complications par l’exérèse complète et méticuleuse évitant les récidives.
